# Genomes of *Candidatus* Wolbachia bourtzisii *w*DacA and *Candidatus* Wolbachia pipientis *w*DacB from the Cochineal Insect *Dactylopius coccus* (Hemiptera: Dactylopiidae)

**DOI:** 10.1534/g3.116.031237

**Published:** 2016-08-19

**Authors:** Shamayim T. Ramírez-Puebla, Ernesto Ormeño-Orrillo, Arturo Vera-Ponce de León, Luis Lozano, Alejandro Sanchez-Flores, Mónica Rosenblueth, Esperanza Martínez-Romero

**Affiliations:** * Centro de Ciencias Genómicas, Universidad Nacional Autónoma de México, Cuernavaca, Morelos, CP 62210, Mexico; † Laboratorio de Ecología Microbiana y Biotecnología “Marino Tabusso”, Universidad Nacional Agraria La Molina, Lima 12, Peru; ‡ Instituto de Biotecnología, Universidad Nacional Autónoma de México, Cuernavaca, Morelos, CP 62210, Mexico

**Keywords:** endosymbiont, scale insect

## Abstract

*Dactylopius* species, known as cochineal insects, are the source of the carminic acid dye used worldwide. The presence of two *Wolbachia* strains in *Dactylopius coccus* from Mexico was revealed by PCR amplification of *wsp* and sequencing of 16S rRNA genes. A metagenome analysis recovered the genome sequences of *Candidatus* Wolbachia bourtzisii *w*DacA (supergroup A) and *Candidatus* Wolbachia pipientis *w*DacB (supergroup B). Genome read coverage, as well as 16S rRNA clone sequencing, revealed that *w*DacB was more abundant than *w*DacA. The strains shared similar predicted metabolic capabilities that are common to *Wolbachia*, including riboflavin, ubiquinone, and heme biosynthesis, but lacked other vitamin and cofactor biosynthesis as well as glycolysis, the oxidative pentose phosphate pathway, and sugar uptake systems. A complete tricarboxylic acid cycle and gluconeogenesis were predicted as well as limited amino acid biosynthesis. Uptake and catabolism of proline were evidenced in *Dactylopius Wolbachia* strains. Both strains possessed WO-like phage regions and type I and type IV secretion systems. Several efflux systems found suggested the existence of metal toxicity within their host. Besides already described putative virulence factors like ankyrin domain proteins, VlrC homologs, and patatin-like proteins, putative novel virulence factors related to those found in intracellular pathogens like *Legionella* and *Mycobacterium* are highlighted for the first time in *Wolbachia*. Candidate genes identified in other *Wolbachia* that are likely involved in cytoplasmic incompatibility were found in *w*DacB but not in *w*DacA.

Many insects possess vertically-transmitted bacterial symbionts that provide them with amino acids and vitamins (Moran 2006). While most insect endosymbionts belong to the Gammaproteobacteria there are others in many other phyla (Moran *et al.* 2008). A remarkable case is the *Wolbachia* endosymbiont that infects between 40% (Zug and Hammerstein 2012) to 66% (Hilgenboecker *et al.* 2008) of arthropod species. *Wolbachia* are phylogenetically affiliated to the Alphaproteobacteria, not distantly related to *Rickettsia*, *Ehrlichia*, and *Anaplasma* (Williams *et al.* 2007). There are 16 phylogenetic supergroups of *Wolbachia* identified, and 10 of them are associated with arthropods (Augustinos *et al.* 2011). Based on phylogenomic analysis, six *Wolbachia* supergroups have been separated in eight species (Ramírez-Puebla *et al.* 2015).

*Wolbachia* are nematode as well as arthropod symbionts (Hilgenboecker *et al.* 2008; Sommer and Streit 2011), and have different effects in their hosts ranging from parasitism to mutualism with spatial and temporal spread of infections in some insect populations (Vavre and Charlat 2012). In nematodes, *Wolbachia* provide vitamins, energy, help in embryo development, and are capable of evading the host immune response (Darby *et al.* 2012; Landmann *et al.* 2014). In arthropods, *Wolbachia* have been found infecting many tissues inside the insect body including reproductive tracts and somatic cells as bacteriocytes (Dobson *et al.* 1999; Clark *et al.* 2005; Hosokawa *et al.* 2010; Sacchi *et al.* 2010; Saha *et al.* 2012). They alter the host reproduction by induction of parthenogenesis (Stouthamer *et al.* 1999), male-killing (Duplouy *et al.* 2013), feminization (Stouthamer *et al.* 1999), and strain incompatibility (Rousset *et al.* 1992). However, it is also known that *Wolbachia* may confer benefits to insects by playing an important role in insect development and survival (Dedeine *et al.* 2001). For example, removal of *Wolbachia* with antibiotics in *Asobara tabida* wasps inhibits maturation of oocytes (Dedeine *et al.* 2001). In *Drosophila*, *Wolbachia* may confer protection against virus infections (Teixeira *et al.* 2008; Chrostek *et al.* 2013) and provide a fecundity benefit to females when subjected to low or high iron diets (Brownlie *et al.* 2009). Thus, *Wolbachia* inside insects may not be only facultative symbionts, but can also be obligate endosymbionts necessary for survival (Dedeine *et al.* 2001).

There are 12 *Dactylopius* species (Ben-Dov 2006; Van Dam and May 2012). Six of them are present in Mexico, including the smallest and most distantly related *Dactylopius tomentosus* (Portillo and Vigueras 2006; Chávez-Moreno *et al.* 2009). *Dactylopius* insects feed exclusively on the sap of cactus plants of the genera *Opuntia* and *Nopalea* (Pérez-Guerra and Kosztarab 1992). Females of these scale insects spend all their lives on the host plant surface, whereas males are winged and short lived. These insects feed on a poor nutritional and low-calorie diet since cactus sap consists mainly of water (88–95%) and is low in nitrogen (0–0.5%) (Stintzing and Carle 2005). The red pigment carmine is obtained from cochineal insects of the genus *Dactylopius*, especially from *D. coccus*, which is a domesticated species. Carmine has been used as a natural dye to color food, medicines, cosmetics, textiles, and artworks, is considered safe for human consumption (Dapson 2005), and has antimicrobial and insecticidal properties (Eisner *et al.* 1980; Pankewitz *et al.* 2007).

Previously, we described a betaproteobacterium, *Candidatus* Dactylopiibacterium carminicum, and other diverse bacterial species associated with *Dactylopius* species present in Mexico (Ramírez-Puebla *et al.* 2010). Here, we extend the knowledge of *Dactylopius* endosymbionts by reporting the presence and genome sequences of two strains of *Wolbachia*, *Candidatus* Wolbachia bourtzisii *w*DacA (supergroup A) and *Candidatus* Wolbachia pipientis *w*DacB (supergroup B) obtained from Mexican *D. coccus*.

## Materials and Methods

### Sample collection

*D. coccus* insects were provided by Campo Carmín Greenhouse (Morelos, Mexico) and were maintained on cactus plants (*Opuntia ficus indica* var. Campo Carmín) in a growth room with controlled photoperiod (12L:12D), temperature (25°), and humidity (40–60%). Other *Dactylopius* species were collected from different states in Mexico: *D. confusus* from Tlaxcala, *D. ceylonicus* from Estado de México, *D. opuntiae* from Querétaro and Mexico City, and *D. tomentosus* from Hidalgo.

### DNA extraction for detection of Wolbachia in Dactylopius individuals

DNA from the whole bodies of adult females of *Dactylopius* species collected in Mexico were extracted and purified with DNeasy Blood & Tissue Kit (QIAGEN) following the manufacturer’s instructions. PCRs were performed using primer pairs wsp81F/wsp691R (Braig *et al.* 1998) and 27F/1492R (Lane 1991) directed to the *wsp* and 16S rRNA genes, respectively.

### Recovery of Wolbachia genomes

Sequence and assembly of metagenomic DNA from samples of pooled *D. coccus* individuals, as well as the recovery of the *Wolbachia* genomes from the metagenome, were previously reported (Ramírez-Puebla *et al.* 2016). For 454 sequencing, 2 g (20 individuals) of adult females were superficially disinfected with 70% ethanol, rinsed with sterile distilled water, and dissected with sterile forceps to remove the exoskeleton and guts. Cells in the hemolymph and debris were separated by centrifugation in a Percoll gradient (adapted from Charles and Ishikawa 1999), phases were observed under a microscope, and those with cells were selected for DNA extraction. For PacBio sequencing, eight individuals were superficially disinfected as previously described. Guts and exoskeleton were removed with sterile forceps. Hemolymph from all individuals was pooled for DNA extraction. For Illumina sequencing, guts, ovaries, and Malpighian tubules from 40 females were dissected using sterile forceps under a stereoscopic microscope. These organs were pooled, suspended in PBS, and macerated using a sterile plastic pestle. In all cases, DNA was extracted with DNeasy Blood & Tissue Kit (QIAGEN) following the manufacturer´s instructions. Sequencing was performed at Macrogen Inc. (Korea) for Illumina and 454 and at Duke University Genome Sequencing Core Facility (USA) for PacBio.

### Genome analysis

The RAST server was used for gene prediction and annotation (Aziz *et al.* 2008). Manual curation of relevant genes was performed after comparisons with sequences deposited in the following databases: nr and Refseq via BLASTX (Benson *et al.* 2013), the Conserved Domain Database at GenBank (http://www.ncbi.nlm.nih.gov/Structure/cdd/wrpsb.cgi), the Protein families (PFAM) database (Finn *et al.* 2014), and the Transport Classification Database (Saier *et al.* 2014). Genome completeness was assessed by the presence of single-copy widespread orthologs with BUSCO (Simão *et al.* 2015). Four out of the forty genes evaluated by BUSCO are absent in all the sequenced *Wolbachia* genomes and this was taken into consideration for the calculations.

### Data availability

The genome sequences of *Wolbachia* strains *w*DacA and *w*DacB have been deposited in the GenBank database under accession numbers LSYX00000000 and LSYY00000000, respectively.

## Results

### Wolbachia in Mexican Dactylopius spp.

Previously, no *Wolbachia* sequences were found by PCR amplification of the 16S rRNA gene with primer pair fD1/rD1 (Weisburg *et al.* 1991) from several *Dactylopius* samples collected in Mexico and Brazil (Ramírez-Puebla *et al.* 2010). We reassessed the presence of these endosymbionts by PCR amplification of the *Wolbachia*-specific *wsp* gene (Braig *et al.* 1998). Amplicons of the expected size were obtained from *D. ceylonicus*, *D. coccus*, *D. confusus*, *D. opuntiae*, and *D. tomentosus*, although not all individuals of each species gave a positive reaction. To identify the *Wolbachia* inhabiting *D. coccus* in Mexico, 16S rRNA gene amplification with primer pair 27F/1492R and sequencing was performed. Two divergent sequences were found; one affiliated with *Wolbachia* supergroup A and the other with supergroup B. Sequences of the latter supergroup were more abundant in all surveyed *D. coccus* individuals (Table 1).

**Table 1:** Abundance of sequences matching *Candidatus* Wolbachia bourtzisii (*w*DacA) and *Candidatus* Wolbachia pipientis (*w*DacB) in *D. coccus* individuals

	Number of 16S rRNA Gene Sequences Assigned to	
Individual	*w*DacA	*w*DacB
Female 1	0	15
Female 2	1	9
Female 3	1	6
Embryo 1	7	8
Embryo 2	2	11

### Divergence between Wolbachia from Dactylopius from different countries

We have recently reported the recovery of two contig bins matching *Wolbachia* from a metagenome of *D. coccus* (Ramírez-Puebla *et al.* 2015, 2016). A phylogenomic analysis of those bins (Ramírez-Puebla *et al.* 2015), confirmed that they corresponded to the genomes of two different *Wolbachia* strains belonging to *Candidatus* Wolbachia bourtzisii (supergroup A) and *Candidatus* Wolbachia pipientis (supergroup B), which will be referred to here as *w*DacA and *w*DacB, respectively.

*Wolbachia* from supergroups A and B were previously reported in *Dactylopius* sp. collected in Lanzarote, Canary Islands, Spain (Pankewitz *et al.* 2007). The Canarian and Mexican *Wolbachia* from supergroups A and B showed, respectively, 99.8% and 98.3% identity at the *ftsZ* gene, and 100% and 98.3% identity at the *wsp* gene. Thus, *Wolbachia* infecting *Dactylopius* sp. populations in the Canary Islands are closely related but distinct to the Mexican *Wolbachia*, the divergence being more pronounced among supergroup B representatives. Recently, a *Wolbachia* genome was recovered during a genome sequencing of *D. coccus* (Campana *et al.* 2015). The reported *w*Coc1 genome was found to belong to supergroup B by *ftsZ* gene sequence analysis, but no analysis of the genome was provided. *w*Coc1 showed 92.4% and 98.2% ANI values with *w*DacA and *w*DacB, respectively, indicating that *w*Coc1 and *w*DacB belong to the same species. No further comparison against our strains was performed because the *w*Coc1 genome assembly was highly fragmented (1064 contigs, N50 size = 1387 bp), and also because that genome may represent a chimera as it is the product of sequences originating from two different and geographically distant *D. coccus* populations, one from Oaxaca in Mexico and the other from Peru.

### Genomes sequences of Wolbachia strains wDacA and wDacB

The number of contigs and N50 sizes of genome assemblies of *w*DacA and *w*DacB were 157 and 13.7 kb and 198 and 14.5 kb, respectively (Table 2), values that were average in comparison to released WGS genomes of *Wolbachia*. Genome completeness assessed with BUSCO (Simão *et al.* 2015) indicated that the recovered genomes of *w*DacA and *w*DacB represented 92% and 94%, respectively, of their whole genomes. It should be pointed out that closed *Wolbachia* genomes are reported as 92–94% complete by BUSCO because from one to three of the evaluated genes are either missing or fragmented in any given genome. Read coverage was widely different between both genomes, 2700 × in *w*DacB *vs.* 174 × in *w*DacA, indicating that the first *Wolbachia* strain is predominant in the tissues of *D. coccus* used in this study. Detection of each *Wolbachia* strain by 16S rRNA gene PCR amplification and sequencing in isolated individuals of *D. coccus* seemed to corroborate that *w*DacB is more abundant than *w*DacA (Table 1). As in other *Wolbachia* strains, *w*DacA and *w*DacB strains showed reduced genomes and low G + C contents (Table 2). Hypothetical genes represented 35% and 23% of the CDS genes in *w*DacA and *w*DacB, respectively. *Wolbachia* strains show high genome plasticity compared with other insect endosymbionts. The presence of a high proportion of mobile DNA and insertion sequences (Bordenstein and Reznikoff 2005; Cordaux *et al.* 2008) may promote this plasticity. The two *Wolbachia* strains of *D. coccus* were not exceptions, although it is worth mentioning that the genome of *w*DacB has a higher number of genes annotated as coding for mobile genetic elements and transposases (404, 24% of the CDS genes) in comparison to *w*DacA (120, 9% of the CDS genes).

**Table 2:** Genome features of *Candidatus* Wolbachia bourtzisii *w*DacA and *Candidatus* Wolbachia pipientis *w*DacB from *D. coccus*

Feature	*w*DacA	*w*DacB	Other *Wolbachia* Genomes [Median (Range)]
Number of contigs	157	198	195 (9 – 1064)*^a^*
N50 (kbp)	13.7	14.5	13.2 (1.4 – 466)*^a^*
Estimated genome size (Mbp)	1.170	1.498	1.224 (0.444 – 1.542)
G + C content (%)	35.1	34.1	34.3 (32.1 – 36.3)
CDS genes	1040	1246	1216 (660 – 1587)*^b^*
With function	720	810	
Hypothetical	320	436	
RNA genes			
rRNA	2	2	3 (0 – 4)*^b^*
tRNA	31	39	34 (14 – 39)*^b^*

*w*DacA, *Candidatus* Wolbachia bourtzisii (supergroup A); *w*DacB, *Candidatus* Wolbachia pipientis (supergroup B); CDS, coding sequence; rRNA, ribosomal RNA; tRNA, transfer RNA.

a Calculated only for unfinished genomes.

b Number of CDS, rRNA, and tRNA genes *de novo* predicted with prodigal, RNAmmer 1.2, and tRNAscan-SE v.1.3.1, respectively.

### Vitamin, coenzymes, cofactors, and nucleotide synthesis

Both *Wolbachia* strains from *D. coccu*s seemed able to synthesize riboflavin and ubiquinone (coenzyme Q). They also had genes required for purine and pyrimidine nucleotide biosynthesis. They lacked complete biosynthesis genes for biotin, thiamine, coenzyme A, NAD, and folic acid. Nevertheless, an uptake system for biotin and a gene for folate salvage were found encoded in each genome. Both strains also possessed a bacterioferritin gene and heme biosynthesis genes.

### Metabolism

The set of genes for the tricarboxylic cycle was complete in both genomes. There were genes for the pentose phosphate pathway but not the oxidative reactions. The phosphofructokinase gene is absent, suggesting that there may be gluconeogenesis but not glycolysis. Cytochrome c oxidase, as well as components of the respiratory complex, were found in both strains.

As has been observed in other *Wolbachia* and other Rickettsiales, most amino acid biosynthesis pathways were incomplete. However, catabolic genes for proline, aspartate, glutamate, and possibly cysteine were identified in both strains. Genes for glutamate dehydrogenase, glutamine synthetase (GS), and glutamate synthase (GOGAT) required for ammonia assimilation were also present. NifU was identified but no other nitrogen fixation genes. Nif proteins involved in the formation of FeS clusters or other metallo clusters can be found in organisms that do not fix nitrogen.

A complete set of genes for fatty acid biosynthesis were present in both genomes as well as for the synthesis of the phospholipids phosphatidylethanolamine, phosphatidylglycerol, and phosphatidylserine. No genes for lipopolysaccharide biosynthesis were found in either genome. *w*DacA and *w*DacB had genes for peptidoglycan synthesis but no transpeptidase genes for chain cross-linking were found.

### Transport

Both genomes encoded genes for ATP-binding cassette (ABC) transporters for uptake of phosphate (*pstABCS* genes), ferric iron, zinc, and possibly lipids; one for export of heme; and one gene for a Mg^+2^ (or Co^+2^) transporter-E (MgtE) family importer. Several genes for putative amino acid symporters were shared by both genomes including five of the major facilitator superfamily (MFS), three of the alanine/glycine:cation symporter (AGCS) family, and one of the dicarboxylate/amino acid:cation symporter (DAACS) family. Strain *w*DacA but not *w*DacB had genes coding for an ABC uptake transporter for glutamine/glutamate. On the other hand, strain *w*DacB possessed three uptake systems of the drug/metabolite transporter (DMT) superfamily, and two genes for organophosphate:phosphate MFS antiporters that were not present in *w*DacA. The former DMT transporters were > 75% similar to the S-adenosylmethionine (SAM) uptake transporter of *Rickettsia prowazekii* (Tucker *et al.* 2003), and the highest similarities (∼49%) of the latter MFS antiporters were to proteins of *R. prowazekii* which have been implicated in triose phosphate uptake used for phospholipid biosynthesis (Frohlich and Audia 2013). No hexose transporter genes were found, supporting the theory that there is no glycolysis in both strains.

Few export transporters were found in both genomes. Besides the heme exporter, both genomes encoded an ABC transporter putatively involved in organic solvent resistance, a CorC-family transporter for magnesium or cobalt efflux, and a cation diffusion-facilitator (CDF) family exporter for zinc or cadmium. In addition, the *w*DacA genome encoded two ABC superfamily transporters, one of the heavy metal transporter (HMT) family related to exporters for phytochelatins-Cd complexes and the other of the multidrug resistance (MDR) family.

### Secretion systems

Of the two systems for protein export into the periplasm, only the general secretion *sec* system was found encoded in *w*DacA and *w*DacB genomes. Protein secretion into the extracellular environment is accomplished by several types of secretion systems, of which only two were found in the *Wolbachia* strains of *Dactylopius*. Both genomes coded for the inner membrane component and the membrane fusion protein of a type I secretion system (T1SS) whose products were 95% and 83% identical, respectively, between the strains. The outer membrane TolC, a channel that acts in conjunction with the other T1SS components, was coded elsewhere in the genomes.

Both strains possessed one type IV secretion system (T4SS). The gene organization was similar to that found in other *Wolbachia* with two separated clusters, one including *virB3*, *virB4*, and four copies of *virB6*, and another cluster with *virB8*, *virB9*, *virB10*, *virB11*, and *virD4*. As it is also observed in other *Wolbachia*, there was one paralogue of each of *virB4*, *virB8*, and *virB9* coded elsewhere in the genomes. Genes *virB1*, *virB2*, *virB5*, *and virB7* have been reported as being absent in *Wolbachia* and in Rickettsiales in general (Pichon *et al.* 2009). However, we found four and three homologs of the pilin *virB2* gene in *w*DacA and *w*DacB, respectively. The *virB2* homologs were not clustered with each other or with other *vir* genes. BLAST searches recovered *virB2* homologs in many *Wolbachia* genomes (data not shown) that are annotated mostly as hypothetical or membrane proteins.

In the symbiotic *w*Bm strain of the nematode *Brugia malayi*, the transcriptional regulators *w*BmxR1 and *w*BmxR2 bind to the promoter regions of some *vir* genes (Li and Carlow 2012). *w*BmxR1 seems to regulate the *virB8* operon (which includes the upstream riboflavin biosynthesis gene *ribA*) and the second copy of *virB9*, while *w*BmxR2 controls the expression of the second copy of *virB4* (Li and Carlow 2012). Homologs coding for proteins > 74% identical to the *w*BmxR1 product were found in both our *Wolbachia* strains, while a homolog to *w*BmxR2 was found only in *w*DacA (78% identity).

### Stress response

Although living in a relatively protected environment inside their host cells, endosymbionts still retain genes required to cope with stressful conditions. Potassium homeostasis is important to react to changes in osmotic pressure and pH changes. One TrkG potassium uptake protein was found encoded in *w*DacA, while there were two in *w*DacB. Both strains had a glutathione-regulated potassium-efflux system KefKL. An HtrA protease/chaperone for degradation of misfolded or mislocalized cell-envelope proteins was encoded in each genome. Genes to contend with oxidative stress, like those for a Fe superoxide dismutase, an alkyl hydroperoxide reductase, three glutaredoxins, and glutathione biosynthesis, were also found. A single gene for a bacterial flavohemoglobin in each genome may be used to contend with nitrosative stress. Common proteins used for temperature stress, such as DnaK-DnaJ-GrpE composing the DnaK chaperone system and GroEL-GroES composing the GroE chaperonin machinery, were found encoded in both genomes as well as a CspA-family cold shock protein. A single sigma factor RpoH protein was encoded in each genome and may be used for stress response.

### Virulence factors

Ankyrin domains are involved in protein–protein interactions and, by interacting with specific regions of the host chromatin, can modulate host gene transcription in other bacteria (Iturbe-Ormaetxe *et al.* 2005; Siozios *et al.* 2013). Genes coding proteins with ankyrin domains were found in both strain genomes, although *w*DacB had double the number of genes in comparison to *w*DacA (34 *vs.* 17 genes). Neither strain possessed genes for chemotaxis or motility via flagella or type IV pilus.

Two of the five MFS transporters of *w*DacA and *w*DacB belonged to the phagosomal nutrient transporter (Pht) family (Chen *et al.* 2008). Amino acid and pyrimidine transporters of this family are required for intracellular survival of *Legionella pneumophila* in macrophages (Sauer *et al.* 2005; Fonseca *et al.* 2014). Homologs to Pht transporters were found encoded in many *Wolbachia* genomes and they were distributed in two major phylogenetic clusters which suggests functional divergence (Supplemental Material, Figure S1).

One gene in each *Wolbachia* strain coded for a protein bearing the mammalian cell entry (MCE) domain. Proteins in this family have been identified as necessary for intracellular colonization and survival by *Mycobacterium tuberculosis* and *M. bovis* (Arruda *et al.* 1993; Flesselles *et al.* 1999). Several genomes of *Wolbachia* (Table S1) as well as other Rickettsiales encoded homologs for MCE proteins. Although not restricted to intracellular bacteria, MCE homologs are present in other facultative or obligate endosymbiotic and parasitic bacteria (Table S2).

Finally, genes coding for proteins that have been highlighted as candidates to induce cytoplasmic incompatibility were also found. Both genomes possessed two copies of the DNA-binding protein HU beta (Beckmann *et al.* 2013). *w*DacB but not *w*DacA had genes coding for proteins that are homologs to WPIP0282, which seems to be present only in *Wolbachia* strains inducing cytoplasmic incompatibility (Beckmann and Fallon 2013). *w*DacB possessed two homologs of the transcriptional regulator *wtrM* gene whose product is able to upregulate the expression of a host gene implicated in cytoplasmic incompatibility (Pinto *et al.* 2013). The *wtrM* gene of *w*DacA was split into two halves by a frameshift mutation.

### Phages

Several genes of gene clusters encoding phage proteins were found in both *Wolbachia* genomes. A complete cluster including phage head-baseplate or head-baseplate-tail genes was not recovered; clusters included either head, baseplate, or tail genes. Paralogous genes located in different head or baseplate clusters in each genome suggested that each strain possesses more than one phage, although it was not possible to determine if any of these phages is complete. The tail clusters, one in each genome, were associated with putative virulence genes: two homologs of the VlrC protein in *w*DacA, and an ankyrin domain protein and a patatin-like protein in *w*DacB.

## Discussion

The presence of *Wolbachia* in *D. coccus* (Campana *et al.* 2015) and *Dactylopius* sp. (Pankewitz *et al.* 2007) has been reported. In this study, *Wolbachia* PCR products were obtained from DNA extracted from Mexican samples of *D. ceylonicus*, *D. coccus*, *D. confusus*, *D. opuntiae*, and *D. tomentosus*. These data show that *Wolbachia* might have started its endosymbiotic state with cochineal insects before the genus *Dactylopius* had diverged.

We report the presence of two *Wolbachia* strains, *w*DacA and *w*DacB, in Mexican individuals of *D. coccus*. The large difference in read coverage between the genomes of *w*DacA and *w*DacB indicates that the latter strain is prevalent in *D. coccus*, at least in the tissue samples used here. Interestingly, *w*DacB but not *w*DacA possessed homologs coding for proteins that are likely involved in causing cytoplasmic incompatibility, a mechanism promoting persistence and dissemination of *Wolbachia* in their hosts. In wasps, double infections of supergroup A and group B strains have been found to influence reproductive and ecological isolation among sibling *Nasonia* species; therefore, *Wolbachia* has been implicated in wasp speciation (Bordenstein *et al.* 2001).

*Dactylopius* insects feed on low-nutrient cactus sap and therefore have to develop strategies to acquire nutrients lacking in their diet from their symbiotic relationships with bacteria. In other insects, riboflavin is produced by their endosymbionts such as the gammaproteobacterium *Wigglesworthia* for Tsetse flies (Akman *et al.* 2002) and *Buchnera* for aphids (Nakabachi and Ishikawa 1999). Recently, it was demonstrated that *Wolbachia* contributes to the growth, survival, and reproduction of bedbugs by riboflavin provisioning (Moriyama *et al.* 2015). Furthermore, it has been postulated that *Wolbachia* strains can also act as heme providers and/or helpers in maintaining iron homeostasis in the host (Brownlie *et al.* 2009; Kremer *et al.* 2009). Both *Wolbachia* strains from *D. coccus* possessed genes for the biosynthesis of riboflavin, heme, and the iron-storage protein bacterioferritin. All these genes are common in *Wolbachia* from insects, even in the sex-manipulator strains that negatively affect the host fitness. For riboflavin, Moriyama *et al.* (2015) have found evidence that its provisioning can counteract the negative effects caused by *Wolbachia* in their hosts.

As has been observed in other *Wolbachia*, *w*DacA and *w*DacB do not have genes to produce most amino acids that their insect hosts require, which may be provided by other bacteria present in *D. coccus* (Ramírez-Puebla *et al.* 2010) that could act as symbionts. The lack of most amino acid biosynthetic capabilities suggests the dependence of *Wolbachia* on its host or on other endosymbionts. Retention of amino acid biosynthesis defines primary insect symbionts and its absence seems to be a characteristic of secondary symbionts (Darby *et al.* 2012). Lack of a functional glycolysis pathway and the presence of several amino acid uptake systems indicate that *Wolbachia* utilizes amino acids instead of sugars as nutrients. Many of the MFS transporters may be proline uptake systems that, together with the presence of PutA for proline catabolism, suggest that this amino acid could be a major nutrient for *Wolbachia*. In fact, high-level expression of PutA has been demonstrated by proteomic analysis of *Wolbachia* (Baldridge *et al.* 2014). Interestingly, proline is an excellent precursor for riboflavin production in the legume endosymbiont *Sinorhizobium* (Phillips *et al.* 1999).

Symbiotic and pathogenic bacteria can use effector proteins delivered to their host via the T4SS to promote intracellular colonization and persistence (Juhas *et al.* 2008). T4SS is widely found in *Wolbachia* strains (Pichon *et al.* 2009) and was also found in *w*DacA and *w*DacB. It was surprising to note that *virB2*, coding for the major T-pilus component, was reported as being absent in *Wolbachia* and other Rickettsiales (Rancès *et al.* 2008; Pichon *et al.* 2009). We found several *virB2* homologs in *w*DacA and *w*DacB, as well as in many *Wolbachia* genomes. This is in agreement with recent data obtained in other Rickettsiales, like *Anaplasma phagocytophilum* (Dugat *et al.* 2014) and *Neorickettsia risticii* (Lin *et al.* 2009) which do possess several *virB2* paralogues. In *N. risticii*, VirB2 is located on the cell surface in agreement with its function as the major T4SS pilus protein (Lin *et al.* 2009). In *A. phagocytophilum*, the AnkA protein is exported via a T4SS (Lin *et al.* 2007). Although T4SS are known to transport proteins and/or DNA, an intriguing possibility is that they can act as nutrient transporters in *Wolbachia* given the scarcity of nutrient export systems in the genomes of these bacteria.

Several efflux systems for heavy metals were found in both genomes suggesting that *Wolbachia* from *D. coccus* have to cope with metal toxicity, perhaps contributed by their host diet. In relation to this, the mucilage of *Opuntia* cacti acts as a good water-soluble chelating polymer (polyelectrolyte) able to remove heavy metals from water (Barka *et al.* 2013), and metal-bound phytochelatin can be found in *Opuntia* shoots (Landero Figueroa *et al.* 2007). Besides heavy metals, other harmful conditions are likely acting on *w*DacA and *w*DacB as both their genomes carried several genes to contend with abiotic stresses. Proteomic profiling of a mosquito *Wolbachia* strain has revealed a profile dominated by chaperones and stress proteins (Baldridge *et al.* 2014).

Another secretion system used by bacteria to interact with eukaryotes is the T1SS. In pathogenic bacteria, virulence factors such as hemolysins are secreted via this system. Secretion of some ankyrin domain proteins by T1SS has been reported in *Rickettsia* (Kaur *et al.* 2012) and *Ehrlichia* (Wakeel *et al.* 2011). Proteins bearing typical T1SS-secretion motifs could not be found in either of our *Wolbachia* genomes, but it is worth noting that several ankyrin domain proteins were coded near the gene for the T1SS inner membrane component in *w*DacB.

In both *Wolbachia* strains, we found transporters of the Pht family, which have been described as virulence factors in *L. pneumophila* (Sauer *et al.* 2005; Fonseca *et al.* 2014). These transporters are present in other Legionellales, in Chlamydiales, as well as in other Rickettsiales besides *Wolbachia*, all having intracellular lifestyles. A protein encoded by *w*DacA and *w*DacB showed homology to the virulence factor Mce of *Mycobacterium* which, when expressed in nonpathogenic *Escherichia coli*, confers the ability to invade and survive within macrophages (Haile *et al.* 2002). The presence of all these putative virulence factors has not been previously pointed out in *Wolbachia*.

## Supplementary Material

Supplemental Material
