# Supplementary material for: Genomes of Candidatus Wolbachia bourtzisii wDacA and Candidatus Wolbachia pipientis wDacB from the Cochineal Insect Dactylopius coccus (Hemiptera: Dactylopiidae)
Source: G3 (Bethesda). 2016 Aug 19;6(10):3343–9. doi: 10.1534/g3.116.031237 (PMC5068953; doi:10.1534/g3.116.031237)
Supplement: Supplemental Material [file supp_6_10_3343__index.html]

Genomes of Candidatus Wolbachia bourtzisii wDacA and Candidatus Wolbachia pipientis wDacB from the Cochineal Insect Dactylopius coccus (Hemiptera: Dactylopiidae) — Supplemental Material 

# Genomes of *Candidatus* Wolbachia bourtzisii *w*DacA and *Candidatus* Wolbachia pipientis *w*DacB from the Cochineal Insect *Dactylopius coccus* (Hemiptera: Dactylopiidae)

## Supplemental Material for Ramirez-Puebla *et al.*, 2016

**Files in this Data Supplement:**

- Figure S1 - Phylogeny of transporters belonging to the Phagosomal nutrient transporter (Pht) family. (.pdf, 46 KB)
- Table S1 - List of *Wolbachia* genomes encoding MCE homologues. (.pdf, 23 KB)
- Table S2 - List of endosymbiotic or parasitic bacteria, besides *Wolbachia*, possessing MCE homologues. (.pdf, 36 KB)
