# Supplementary material for: Genomes of Candidatus Wolbachia bourtzisii wDacA and Candidatus Wolbachia pipientis wDacB from the Cochineal Insect Dactylopius coccus (Hemiptera: Dactylopiidae)
Source: G3 (Bethesda). 2016 Aug 19;6(10):3343–9. doi: 10.1534/g3.116.031237 (PMC5068953; doi:10.1534/g3.116.031237)
Supplement: Supplemental Material [file supp_g3.116.031237_FigureS1.pdf]

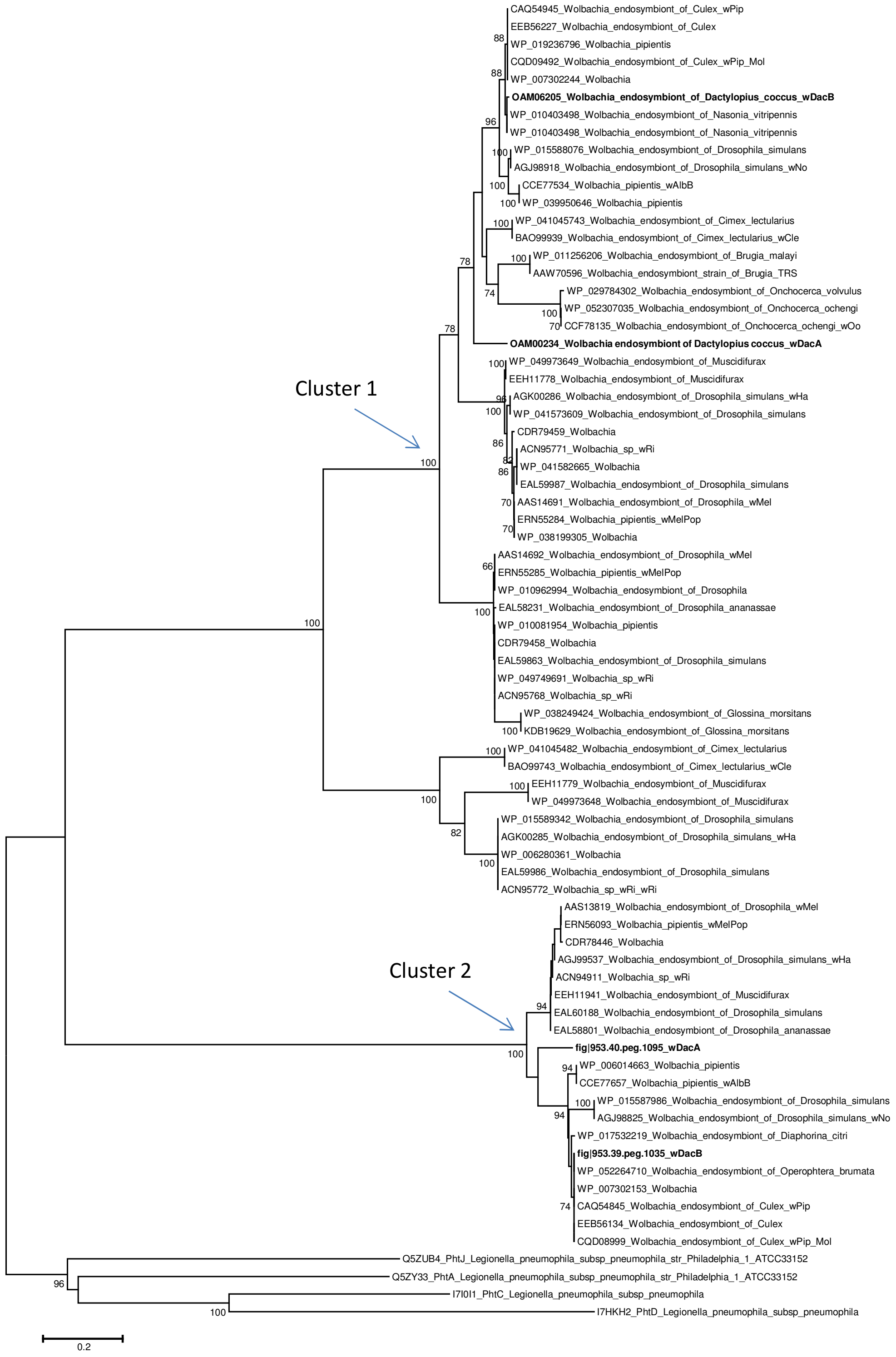

Figure S1. Phylogeny of transporters belonging to the Phagosomal nutrient transporter (Pht) family. Clusters 1 and 2 include only *Wolbachia* sequences.
