# Supplementary material for: Genomes of Candidatus Wolbachia bourtzisii wDacA and Candidatus Wolbachia pipientis wDacB from the Cochineal Insect Dactylopius coccus (Hemiptera: Dactylopiidae)
Source: G3 (Bethesda). 2016 Aug 19;6(10):3343–9. doi: 10.1534/g3.116.031237 (PMC5068953; doi:10.1534/g3.116.031237)
Supplement: Supplemental Material [file supp_g3.116.031237_TableS1.pdf]

Table S1. List of *Wolbachia* genomes encoding MCE homologues.

Strain	Supergroup	Host
*w*Ana	A	*Drosophila ananassae*
*w*Ri	A	*Drosophila simulans*
*w*Ha	A	*Drosophila simulans*
*w*Uni	A	*Muscidifurax uniraptor*
*w*Mel	A	*Drosophila melanogaster*
*w*MelPop	A	*Drosophila melanogaster*
*w*Au	A	*Drosophila simulans*
*w*Gmm	A	*Glossina morsitans morsitans*
*w*VitB	B	*Nasonia vitripennis*
*w*AlbB	B	*Aedes albopictus*
*w*Pip Pel	B	*Culex quinquefasciatus*
*w*Pip JHB	B	*Culex quinquefasciatus*
*w*Pip Mol	B	*Culex molestus*
*w*No	B	*Drosophila simulans*
*w*Di	C	*Diaphorina citri*
*w*Bm	D	*Brugia malayi*
*w*Cle	F	*Cimex lectularius*
