# Supplementary material for: Genomes of Candidatus Wolbachia bourtzisii wDacA and Candidatus Wolbachia pipientis wDacB from the Cochineal Insect Dactylopius coccus (Hemiptera: Dactylopiidae)
Source: G3 (Bethesda). 2016 Aug 19;6(10):3343–9. doi: 10.1534/g3.116.031237 (PMC5068953; doi:10.1534/g3.116.031237)
Supplement: Supplemental Material [file supp_g3.116.031237_TableS2.pdf]

Table S2. List of endosymbiotic or parasitic bacteria, besides *Wolbachia*, possessing MCE homologues.

Genus/species	Order	Class	Host	Bacteria described in
Caedibacter varicaedens	Rickettsiales	Alphaproteobacteria	*Paramecium aurelia*	(Quackenbush 1978)
Candidatus Arcanobacter lacustris	Rickettsiales	Alphaproteobacteria	Unknown	(Martijn *et al.* 2015)
Endozoicomonas montiporae	Oceanospirillales	Gammaproteobacteria	*Montipora aequituberculata*	(Yang *et al.* 2010)
Candidatus Midichloria mitochondrii	Rickettsiales	Alphaproteobacteria	Mitochondria of ovarian cells of *Ixodes ricinus*	(Beninati *et al.* 2004)
Endosymbiont of *Acanthamoeba* sp.	Rickettsiales	Alphaproteobacteria	*Acanthamoeba* sp.	Unpublished
Candidatus Endolissoclinum faulkneri	Rhodospirillales	Alphaproteobacteria	*Lissoclinum patella*	(Kwan *et al.* 2012)
Micavibrio aeruginosavorus	Unclassified	Alphaproteobacteria	Various	(Davidov *et al.* 2006)

References

Beninati, T., N. Lo, L. Sacchi, C. Genchi, H. Noda *et al.*, 2004 A novel alpha-Proteobacterium resides in the mitochondria of ovarian cells of the tick *Ixodes ricinus*. Applied and Environmental Microbiology 70: 2596-2602.

Davidov, Y., D. Huchon, S.F. Koval, and E. Jurkevitch, 2006 A new α-proteobacterial clade of *Bdellovibrio*-like predators: implications for the mitochondrial endosymbiotic theory. Environmental Microbiology 8: 2179-2188.

Kwan, J.C., M.S. Donia, A.W. Han, E. Hirose, M.G. Haygood *et al.*, 2012 Genome streamlining and chemical defense in a coral reef symbiosis. Proceedings of the National Academy of Sciences 109: 20655-20660.

Martijn, J., F. Schulz, K. Zaremba-Niedzwiedzka, J. Viklund, R. Stepanauskas *et al.*, 2015 Single-cell genomics of a rare environmental alphaproteobacterium provides unique insights into Rickettsiaceae evolution. ISME Journal 9: 2373-2385.

Quackenbush, R.I., 1978 Genetic relationships among bacterial endosymbionts of *Paramecium aurelia*. Microbiology 108: 181-187.

Yang, C.S., M.H. Chen, A.B. Arun, C.A. Chen, J.T. Wang *et al.*, 2010 *Endozoicomonas montiporae* sp. nov., isolated from the encrusting pore coral *Montipora aequituberculata*. International Journal of Systematic and Evolutionary Microbiology 60: 1158-1162.
